# The Passive Monitoring of Depression and Anxiety Among Workers Using Digital Biomarkers Based on Their Physical Activity and Working Conditions: 2-Week Longitudinal Study

**DOI:** 10.2196/40339

**Published:** 2022-11-30

**Authors:** Kazuhiro Watanabe, Akizumi Tsutsumi

**Affiliations:** 1 Department of Public Health Kitasato University School of Medicine Sagamihara Japan

**Keywords:** digital biomarkers, mobile health, mental health, psychological distress, depression, anxiety, physical activity

## Abstract

**Background:**

Digital data on physical activity are useful for self-monitoring and preventing depression and anxiety. Although previous studies have reported machine or deep learning models that use physical activity for passive monitoring of depression and anxiety, there are no models for workers. The working population has different physical activity patterns from other populations, which is based on commuting, holiday patterns, physical demands, occupations, and industries. These working conditions are useful in optimizing the model used in predicting depression and anxiety. Further, recurrent neural networks increase predictive accuracy by using previous inputs on physical activity, depression, and anxiety.

**Objective:**

This study evaluated the performance of a deep learning model optimized for predicting depression and anxiety in workers. Psychological distress was considered a depression and anxiety indicator.

**Methods:**

A 2-week longitudinal study was conducted with workers in urban areas in Japan. Absent workers were excluded. In a daily survey, psychological distress was measured using a self-reported questionnaire. As features, activity time by intensity was determined using the Google Fit application. Additionally, we measured age, gender, occupations, employment status, work shift types, working hours, and whether the response date was a working or nonworking day. A deep learning model, using long short-term memory, was developed and validated to predict psychological distress the next day, using features of the previous day. Further, a 5-fold cross-validation method was used to evaluate the performance of the aforementioned model. As the primary indicator of performance, classification accuracy for the severity of the psychological distress (light, subthreshold, and severe) was considered.

**Results:**

A total of 1661 days of supervised data were obtained from 236 workers, who were aged between 20 and 69 years. The overall classification accuracy for psychological distress was 76.3% (SD 0.04%). The classification accuracy for severe-, subthreshold-, and light-level psychological distress was 51.1% (SD 0.05%), 60.6% (SD 0.05%), and 81.6% (SD 0.04%), respectively. The model predicted a light-level psychological distress the next day after the participants had been involved in 3 peaks of activity (in the morning, noon, and evening) on the previous day. Lower activity levels were predicted as subthreshold- and severe-level psychological distress. Different predictive results were observed on the basis of occupations and whether the previous day was a working or nonworking day.

**Conclusions:**

The developed deep learning model showed a similar performance as in previous studies and, in particular, high accuracy for light-level psychological distress. Working conditions and long short-term memory were useful in maintaining the model performance for monitoring depression and anxiety, using digitally recorded physical activity in workers. The developed model can be implemented in mobile apps and may further be practically used by workers to self-monitor and maintain their mental health state.

## Introduction

Physical activity is an important health-related bodily activity for treating and preventing depression and anxiety [1]. Daily physical activity protects against depressive moods experienced by individuals in their daily lives [2]. In the working population, physical inactivity is widespread [3] owing to an increase in work involving low-intensity activities [4]. Based on its effectiveness in preventing depression and anxiety, which are common among workers [5,6], promoting physical activity is the most obvious intervention to effectively prevent common mental disorders in the workplace [7]. For healthy working people, information on their physical activity is useful to self-monitor and maintain their mental health.

Recent studies have shown that physical activity measured using digital tools serve as digital biomarkers in the passive monitoring of depression and anxiety. Furthermore, novel digital technologies and machine or deep learning models have used physical activity to predict depression and anxiety [8-19]. A recent systematic review [8] reported that 19 studies measured physical activity using smartphones and wearable devices to passively monitor depression. It was discovered that activity time, level, intensity, movement speed, and step counts were indicators that are significantly correlated with depression. These indicators predominantly negatively correlated with depression. Support vector machines [9,10], random forests [11,12], regression trees [15,16], regression models [13,18], and ensemble learning were used as learning models. The predictive performance of these models showed moderate to strong correlations with depression and approximately 80% accuracy for depression severity.

However, no studies have developed models that use working conditions to passively monitor depression and anxiety in workers by measuring physical activity using digital tools. A previous study [18] sampled workers and used physical activity, sleep, and the heart rate as features. However, the study did not use information on the working conditions. Other models did not target workers but were based on college or undergraduate students or patients. Regarding the employed population, patterns of physical activity reflected the differences between other populations and that within the population based on commuting or holiday patterns, physical demands, occupations, and industries [20]. These working conditions would explain the variations in physical activities and was useful in optimizing the model to monitor depression and anxiety in workers. Additionally, deep learning models, such as recurrent neural networks, which have feedback connections with successive inputs, would increase the prediction accuracy because the information in the previous inputs regarding physical activity and the state of depression and anxiety explain subsequent inputs.

This study aims to evaluate the predictive and classification performance of a deep learning model for analyzing depression and anxiety, that is, psychological distress, which has been optimized for workers. We used workers’ physical activity time (measured using a smartphone app) and the psychological distress state from the previous day as features to monitor their psychological distress on the next day. Additionally, working conditions (information on whether the previous day was a working day or not), occupation, employment status, shift type, and working hours were considered features. The long short-term memory (LSTM) model was used as a deep learning model. We hypothesized that the deep learning model developed using the abovementioned characteristics would have similar or better classification performances than models used in previous studies [8-19]. Additionally, the model is expected to have a strong correlation with the measured levels of psychological distress. This study is the first investigation to develop an optimized model using working conditions and LSTM to predict depression and anxiety in workers, and it could be useful to workers to self-monitor and be a primary prevention from depression and anxiety.

## Methods

### Study Design and Settings

A 2-week longitudinal study with workers was conducted from November 2021 to April 2022 to measure their daily psychological distress and obtain digital data on their physical activity and working conditions. Participating workers in Japan were recruited from private companies in the Kanto region and the social networking platform Twitter. Recruitments, collection of informed consent, and data collection, which included conducting surveys, were carried out digitally via email. The following were the eligibility criteria for the study participants: (1) working in a public- or private-sector organization, (2) living or working in urban areas, and (3) having a personal smartphone. We excluded workers who were absent at baseline.

A total of 236 workers, aged between 20 and 69 years, participated in this study. Table 1 presents the characteristics of the participants.

Initially, the participants were asked to complete a baseline survey to assess their working conditions. The first page of the baseline survey explained the terms and conditions of the study, which participants had to read and approve before proceeding to the next page. Additionally, they were asked to install the Google Fit app [21,22] (available on both Android and iOS) on their smartphones. During the observational study period, surveys were distributed from 5 to 6 PM daily to measure the psychological distress of the participants and check whether the response was provided on a working or nonworking day. After this 2-week period, the participants exported and sent their Google Fit app data to us.

**Table 1:** Characteristics of participants at baseline (N=236).

Characteristics			Participants, n (%)
**Age (years)**			
	<20	0 (0)	
	20-29	40 (16.9)	
	30-39	72 (30.5)	
	40-49	64 (27.1)	
	50-59	53 (22.5)	
	60-69	7 (3.0)	
	≥70	0 (0)	
**Gender**			
	Male	132 (55.9)	
	Female	104 (44.1)	
	Others or not responded	0 (0)	
**Occupation**			
	Managers	45 (23.3)	
	Professions, engineers, or academics	68 (27.9)	
	Clerks	64 (23.3)	
	Sales	27 (11.4)	
	Services	14 (5.9)	
	Transportation	2 (0.8)	
	Construction	0 (0)	
	Production/Skilled	5 (2.1)	
	Agriculture/Forestry/Fisheries	0 (0)	
	Others	11 (4.7)	
**Employment status**			
	Full-time	202 (85.6)	
	Part-time	12 (5.1)	
	Dispatched	3 (1.3)	
	Contract	10 (4.2)	
	Others	9 (3.8)	
**Shift type**			
	Day shift	223 (94.5)	
	Rotation shift	7 (2.9)	
	Night shift	0 (0)	
	Others	6 (2.5)	
**Working hours (per week; hours)**			
	1-34	21 (8.9)	
	35-40	60 (25.4)	
	41-50	114 (48.3)	
	51-60	34 (14.4)	
	61-65	4 (1.7)	
	66-70	1 (0.4)	
	≥71	2 (0.8)	

### Measurement of Supervised Data

#### Psychological Distress

In the daily survey, psychological distress of the participants was measured using the Japanese version of the K6 scale [23]. The K6 scale comprises 6 items that assess how often people experience symptoms of depression and anxiety; these items were rated on a 5-point Likert scale (0=none of the times and 4=all the times). The reliability and validity of the Japanese K6 scale was confirmed [23], and the internal consistency in this study was quite high (Cronbach α=.92). In this study, the total score on the K6 scale was considered a psychological distress indicator. Additionally, the participants were divided into 3 groups based on the cutoff scores under the K6 scale: light level (<5), subthreshold level (≥5 and <13), and severe level (≥13) [24,25]. This grouping was used to evaluate the classification performance of the deep learning model.

#### Physical Activity

Digital data on physical activity were obtained using the Google Fit app. Based on a previous systematic review [8], we adopted activity time by intensity as a physical activity feature. The Google Fit app could store 24 h of physical activity every 15 minutes in accordance with intensity (light and moderate to vigorous). Details of the measurement methodology and reliability of the data are described elsewhere [26]. The app used smartphone sensors or a heart rate monitor to track the physical activities of the workers and categorize the intensity of these activities on the basis of their heart rate. The maximum heart rate was calculated using the following formula: maximum heart rate=205.8–(0.685×age). If the heart rate was ≥50% that of the maximum value, the activity was categorized as vigorous.

#### Working Conditions

As an additional feature, working conditions were measured under the baseline and daily surveys. In the baseline survey, we obtained data on the age (<20, 20-29, 30-39, 40-49, 50-59, 60-69, and ≥70 years), gender (male, female, others, and ones with no response), type of occupation (management, engineering or education, general office tasks, sales, services, transportation, construction, and production or skill, and agriculture, forestry, or fisheries), employment status (full-time, part-time, dispatched, and contract), work shift types (day shift, rotation, and night shift), and working hours per week (1-34, 35-40, 41-50, 51-60, 61-65, 66-70, and ≥71 hours). In the daily survey, we measured whether the response date was that of a working or nonworking day.

### Analysis

#### Procedure for Validation of Deep Learners

**Figure 1:**
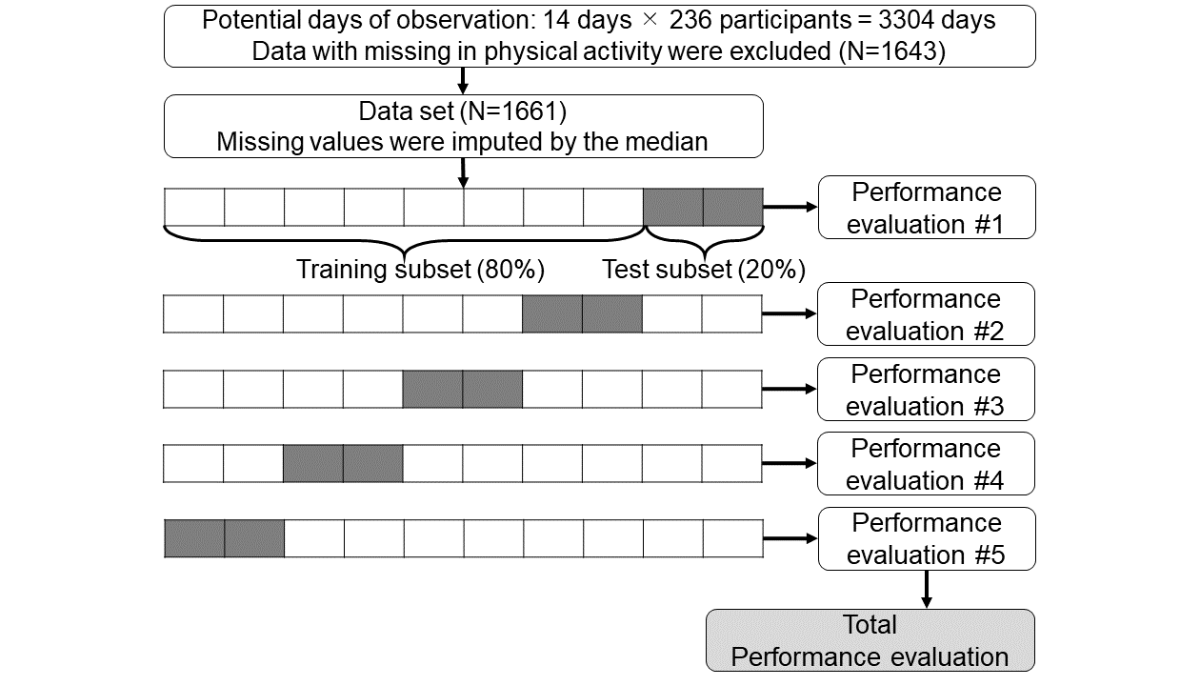
Procedure for developing, validating, and evaluating the deep learning algorithms.

A deep learning model was developed to predict the log-transformed psychological distress in the participants on the day after their features were collected. Figure 1 shows the procedure for developing, validating, and evaluating the deep learning algorithms. A total of 1661 supervised data were used for validating and evaluating the model, which comprised data from the previous day (physical activity, working conditions, and psychological distress) and data from the next day (psychological distress). Of potential days of observation among 236 participants in the 2 weeks (3304 days), data with missing information on physical activity were excluded because the participants could not obtain their Google Fit app data. Missing values in the daily survey were imputed using medians.

A 5-fold cross-validation method was used to develop and evaluate the deep learning algorithm, using the K-Folds cross-validator in scikit-learn. The daily supervised data were randomly divided into 5 subsets, and each subset was used as the training and test data sets in rotation. Performance evaluation was conducted for each trained model using the test subsets, and the overall model evaluation was calculated as an average of the 5 performance scores.

As the deep learning model, the LSTM model was used, which is a recurrent neural network model that has feedback connections for handling consecutive inputs [27], such as the activity times in the previous periods. The length of the input was set to 96, which was equal to the length of the data on the physical activity of a worker for a single day (24 hours for every 15 minutes). Scores of K6 from the daily survey and information on the working conditions at the baseline survey were expanded to 96 lengths to align with the length of the physical activity. Therefore, the shape of the input data was a 3-rank tensor: the number of observational days (n=1661), 96, and the number of features (n=17). Occupations were one-hot encoded. The output was a log-transformed score on the K6 scale on the next day. The number of hidden layers in the LSTM model was set to 27. Node and recurrent dropout rates were set at 20%. Regarding the activation functions, the rectified linear unit, sigmoid, and linear activations were used as the hidden, recurrent, and output layers, respectively. The Adam optimization algorithm was adopted [28] with a learning rate of 0.0002. The mean squared error was used as the loss function. The batch size was set to 10, and the epoch number was set to 250. To avoid overfitting, early stopping was implemented when the loss values for the current epoch exceeded those for the 10 previous epochs. Data handling was implemented using NumPy (version 1.19.4) [29] and scikit-learn (version 0.23.1) [30]. The model development, training, and validation processes were implemented using Keras (version 2.6.0) [31].

#### Model Evaluation

As the primary indicator of performance, classification accuracy for the severity of the psychological distress was considered: light level (<5), subthreshold level (≥5 and <13), and severe level (≥13). As secondary indicators, the Pearson correlation coefficient (*r*) and *R*^2^ values were also calculated between the predicted and measured values for psychological distress.

#### Model Interpretation

To interpret how the deep learning model predicted and classified the scores and severity of psychological distress, the means of the activity time by intensity were depicted and stratified in accordance with the classification results (light, subthreshold, or severe level) in the training data.

### Ethical Considerations

The study protocol was approved by the Kitasato University Medical Ethics Organization (B21-119). Informed consent was obtained before the baseline survey. On the web page, potential participants were asked to read and approve the terms and conditions of the study before proceeding to the baseline survey page. The terms and conditions stated that the research team would protect the privacy and confidentiality of the collected data and that the study data would be deidentified before analyses. The participants did not receive any compensation in this study.

## Results

### Performance Evaluation of the Deep Learning Model

A total of 1661 days of supervised data were obtained from 236 participants. The mean K6 score was 2.78 (SD 4.27). A total of 131 days of missing values for the K6 scores and the type of day (ie, working or nonworking) were imputed using the median. The median of K6 score was 1, and the median type of day was working. The cross-validation method divided the data into 5 sub–data sets: 4 subsets comprised 332 days of data and the others comprised 333 days of data. Of these, 96.40 (SD 6.0) days (29.02%, SD 1.8%) were nonworking days. For the learning process, 11 to 81 epochs were required to complete the process (Multimedia Appendix 1). The mean squared error values of the training data during the training and validation of the deep learning model gradually declined as the number of epochs increased. The mean score of the mean squared error of the test data was 0.386 (SD 0.06).

Table 2 shows the matrix of the predicted and measured classifications of psychological distress severity in the 5 test sub–data sets. The overall categorization accuracy of the deep learning model was 76.3% (SD 0.04%). The classification accuracies for the severe-, subthreshold-, and light-level psychological distress were 51.1% (SD 0.05%), 60.6% (SD 0.05%), and 81.6% (SD 0.04%), respectively. The correlation coefficient between the predicted and measured values for psychological distress was 0.679 (SD 0.05; *R*^2^=0.463, SD 0.07).

**Table 2:** Classification performance of the 5 test sub–data sets.

Number of classified data (sub–data sets #1, #2, #3, #4, and #5)				Measured psychological distress (days), n							Total (days), n
				Severe (K6 score≥13)		Subthreshold (K6 score≥5 and <13)		Light (K6 score<5)			
**Predicted psychological distress**											
	**Severe (K6 score≥13)**										
		Sub–data set 1	*8* ^a^		3		2		13		
		Sub–data set 2	*7*		5		0		12		
		Sub–data set 3	*11*		9		2		22		
		Sub–data set 4	*5*		7		0		12		
		Sub–data set 5	*7*		4		1		12		
	**Subthreshold (K6 score≥5 and <13)**										
		Sub–data set 1	7		*39*		59		105		
		Sub–data set 2	3		*44*		41		88		
		Sub–data set 3	5		*32*		43		80		
		Sub–data set 4	4		*44*		36		84		
		Sub–data set 5	5		*33*		49		87		
	**Light (K6 score<5)**										
		Sub–data set 1	3		24		*188*		215		
		Sub–data set 2	3		21		*208*		232		
		Sub–data set 3	3		16		*211*		230		
		Sub–data set 4	2		13		*221*		236		
		Sub–data set 5	1		22		*210*		233		
**Total**											
	Sub–data set 1			18		66		249		333	
	Sub–data set 2			13		70		249		332	
	Sub–data set 3			19		57		256		332	
	Sub–data set 4			11		64		257		332	
	Sub–data set 5			13		59		260		332	

^a^Cells with italicized values indicate accurate classification.

### Model Interpretation

**Figure 2:**
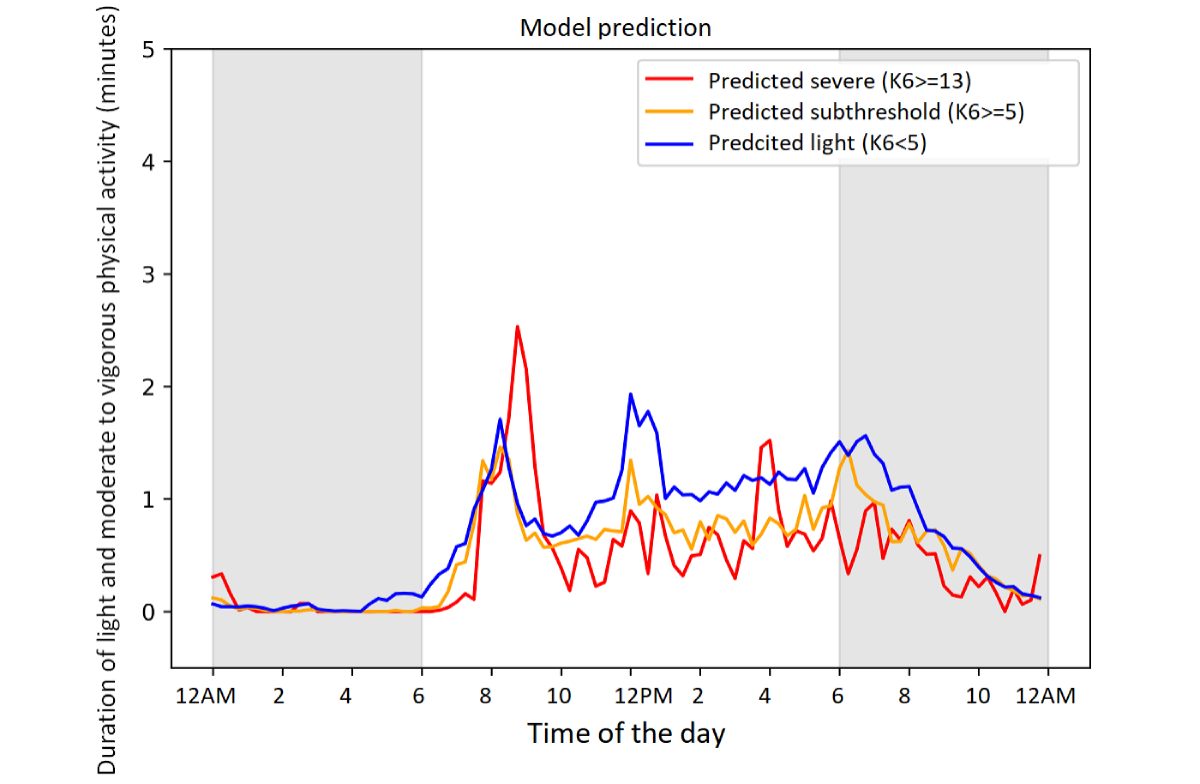
Duration of light and moderate to vigorous physical activity (minutes) within a day stratified by the severity of psychological distress.

Figure 2 shows the mean of the activity time (in minutes) of light and moderate to vigorous physical activity stratified in accordance with the prediction of the psychological distress severity. The developed deep learning model predicted a light-level psychological distress on the next day when the participants had 3 peaks of activity (from 10 to 20 minutes) in a day; that is, in the morning (8 AM), at noon (12 PM), and in the evening (6 PM). The patterns that were predicted as the subthreshold and severe levels of psychological distress followed a similar trend. However, several levels of activity were relatively lower than light distress levels. The pattern predicted for the severe psychological distress level showed a longer activity time in the morning.

The mean of the activity time, which was stratified in accordance with working conditions, showed different patterns based on the prediction of the psychological distress severity. The activity time on a working day showed a similar trend to the overall results. Contrarily, those on a nonworking day exhibited very different results (Multimedia Appendix 2). On the one hand, the activity time patterns predicted for light level psychological distress had a smaller peak for the activity times in the morning and a plateau in the afternoon rather than those on a working day. On the other hand, shifted peaks in the morning (9 AM) and afternoon (4 PM) were predicted as indicators for a severe level of psychological distress the next day. Differences in activity-time patterns were also observed when stratified in accordance with occupations (Multimedia Appendix 3). The 3 predicted peaks for the activity time were similar when predicted as a light level of psychological distress. However, spikes among managers, sales, and service workers were predicted as subthreshold- and severe-level psychological distress. Moreover, numerous peaks were observed during the day for workers engaged in service occupations. Among them, levels of activity were relatively higher when patterns were predicted as the subthreshold level of psychological distress than those of light-level distress.

## Discussion

### Principal Findings

The deep learning model developed using LSTM, based on the physical activities and working conditions, revealed a similar performance as reported in previous studies [8-19]. The accuracy in classifying the severity of psychological distress was high, indicating a relatively high performance in classifying light-level distress. The predicted values for psychological distress correlated strongly with the measured values, and over 45% of the variance in distress was explained. The results suggest that the performance was maintained using the information on the working conditions and model, which had feedback connections to process a range of information, even when other different digital biomarkers were not considered (eg, sleep, heart rate, sociability, location, and smartphone use). Working conditions and LSTM were useful in maintaining the model’s performance for monitoring depression and anxiety by using digitally recorded physical activities in workers.

The prediction and classification performance of the deep learning model was similar to that in a previous study [18] that targeted a working population. A significant advantage of the proposed model was its high accuracy for detecting light-level psychological distress, which implies that the model accurately assessed mentally healthy workers on the basis of the features obtained. In other words, workers can take care of their physical activities to prevent mental disorders before they even occurred, by using the model. This was useful for workers to maintain their physical activities and monitor whether the activity time patterns were good for their mental health. Good patterns showed 3 peaks of 10-20 minutes of physical activity in the morning, during midday, and in the evening. These patterns reflect arrival and departure from work and lunch and closely resembled those previously surveyed among workers in Singapore [18], which suggests that these patterns represent a basic and healthy rhythm among workers. Contrarily, lower levels of physical activity were associated with subthreshold- and severe-level psychological distress. These findings correspond with those of previous studies, which showed that the digital biomarkers of physical activity were predominantly negatively correlated with depression [8]. The patterns classified as severe-level psychological distress showed no peak in the noon and evening; this might reflect low-level activity owing to long working hours or high workloads. The activity patterns reflected workers’ rhythm of life, which was influenced not only by their own behaviors but also factors at work.

Interestingly, there were predictive differences in working conditions. Shiftable peaks in the later activities and high levels of activity on nonworking days led to high psychological distress. Additionally, excessive workload peaks among managers, sales, and service workers were predicted to be subthreshold- and severe-level psychological distress, respectively. On the one hand, among managers, physical activity at night (after 8 PM) might be related to the severe-level distress. Service workers had several peaks of physical activity, and higher-level peaks were predicted as subthreshold-level distress. On the other hand, clerks and professionals did not have a similar trend: lower whole levels of physical activities were associated with subthreshold- and severe-level psychological distress. These differences might depend on the activity levels of their demanding work. Managerial, sales, and service jobs are qualitatively different from clerical and professional jobs and tend to be more physically demanding. Excessive physical activity could affect the psychological distress of workers if the activities at work are physically demanding. These findings may be attributed to poor sleep and circadian rhythms [32-34], long working hours [35,36], or the physical demands of work activity [37]. A previous study showed that nighttime heart rate variations were the most important feature in their machine learning model, and discussed the circadian and rest rhythms as possible underlying mechanisms [18].

The classification performance of the developed model for the severe-level psychological distress was not high. Misclassified samples included more data from rotation and other shift workers (12%) than from the whole sample. Workers who were engaged in shift work had a different rhythm from day-shift workers. Hence, they needed different algorithms to predict the level of psychological distress. This study did not cover much data on shift workers. Consequently, further studies are needed to tune the model.

### Limitations

Several limitations limited the validity and generalizability of the study. The lack of numbers and variations in the data monitored led to low generalizability. Particularly, severely distressed night-shift workers were lacking. The results could not be directly compared with those of previous studies because the latter used different measurements and digital biomarkers. Previous studies surveyed students and patients and mainly used the Patient Health Questionnaire as an indicator for depression. Information on sleep, an important digital biomarker used in the previous studies [14,17,18], was measured in this study. Daily information on the working hours and workloads were lacking in this study, which contributed considerably to predicting psychological distress. Some participants did not have their smartphones with them during work hours because of their work preferences or rules, thereby leading to a biased estimate regarding their activity time.

### Conclusions

In conclusion, the developed deep learning model performed similarly to those reported in previous studies and had high accuracy in determining light-level psychological distress as a function of physical activity and working conditions. The collected information on the working conditions was useful in passively monitoring the depression and anxiety status of workers. It further contributed in determining the mental health status of workers by using digital biomarkers. The developed model can be used in mobile apps and among workers to self-monitor and maintain their mental health state.

## Appendix

Multimedia Appendix 1 Mean squared error as a loss function during training in the five test subsets.

Multimedia Appendix 2 Duration of light and moderate to vigorous physical activity (minutes) within a day stratified by the severity of psychological distress for working and non-working days.

Multimedia Appendix 3 Duration of light and moderate to vigorous physical activity (minutes) within a day stratified by the severity of psychological distress for different occupations.

## Abbreviations

LSTM: long short-term memory

## Data Availability

The data sets generated and analyzed in this study are available with the corresponding author upon reasonable request.
