# Supplementary figures and images for: The Passive Monitoring of Depression and Anxiety Among Workers Using Digital Biomarkers Based on Their Physical Activity and Working Conditions: 2-Week Longitudinal Study
Source: JMIR Form Res. 2022 Nov 30;6(11):e40339. doi: 10.2196/40339 (PMC9752468; doi:10.2196/40339)

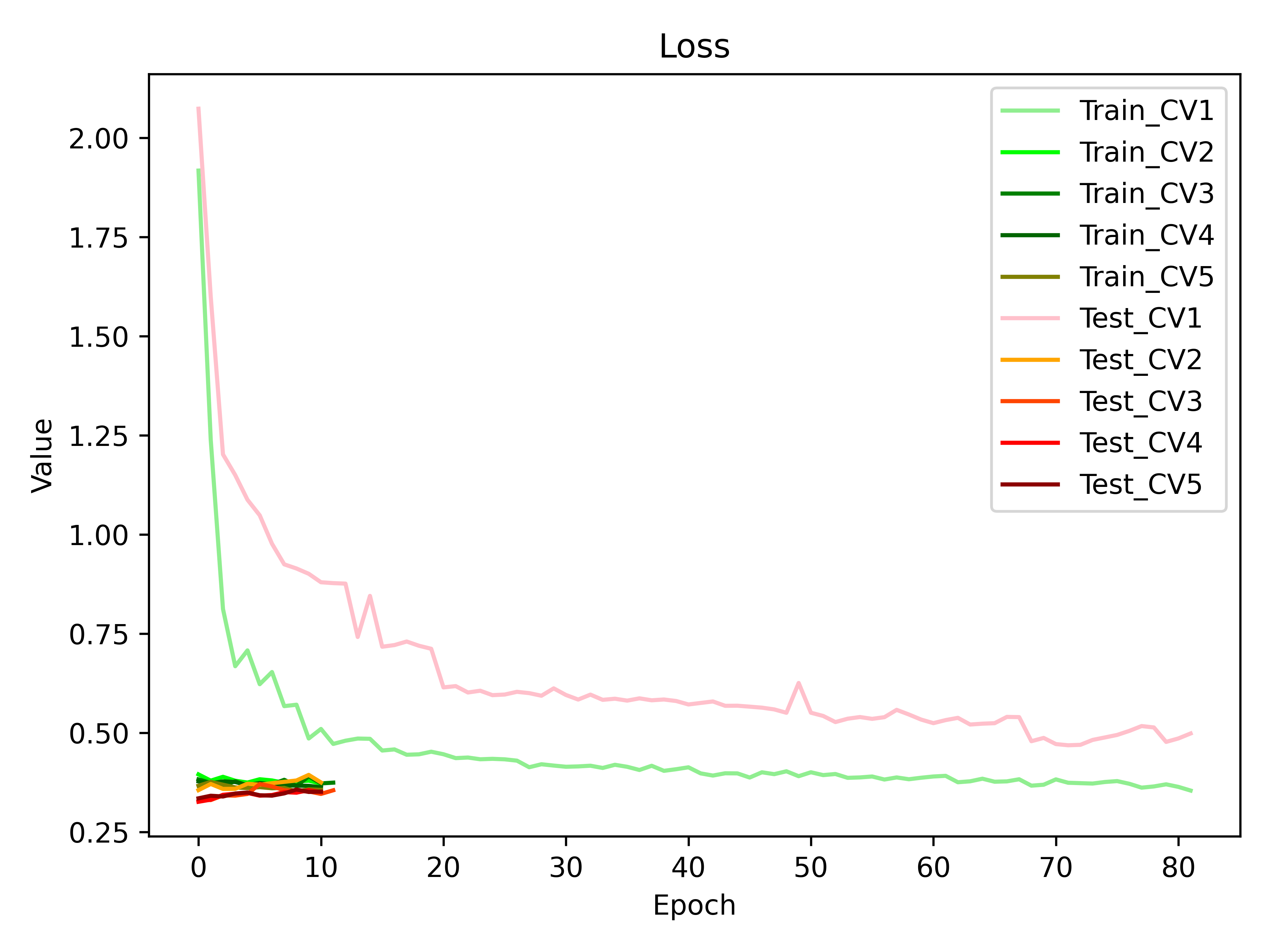

Supplement: Multimedia Appendix 1 [file formative_v6i11e40339_app1.png]

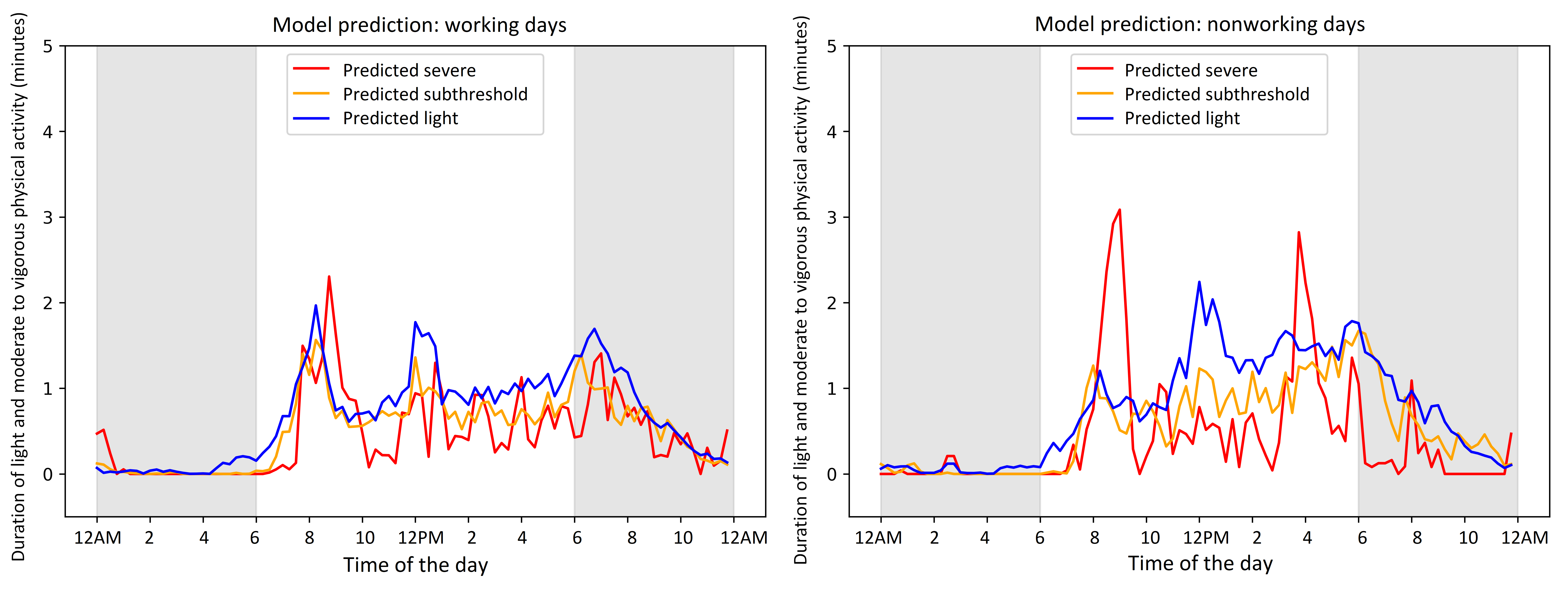

Supplement: Multimedia Appendix 2 [file formative_v6i11e40339_app2.png]
